# Mining EEG with SVM for Understanding Cognitive Underpinnings of Math Problem Solving Strategies

**DOI:** 10.1155/2018/4638903

**Published:** 2018-01-11

**Authors:** Paul Bosch, Mauricio Herrera, Julio López, Sebastián Maldonado

**Affiliations:** ^1^Facultad de Ingeníera, Universidad del Desarrollo, Av. Plaza 700, Las Condes, Santiago, Chile; ^2^Facultad de Ingeniería y Ciencias, Universidad Diego Portales, Ejército 441, Santiago, Chile; ^3^Facultad de Ingeniería y Ciencias Aplicadas, Universidad de los Andes, Monseñor Álvaro del Portillo 12455, Las Condes, Santiago, Chile

## Abstract

We have developed a new methodology for examining and extracting patterns from brain electric activity by using data mining and machine learning techniques. Data was collected from experiments focused on the study of cognitive processes that might evoke different specific strategies in the resolution of math problems. A binary classification problem was constructed using correlations and phase synchronization between different electroencephalographic channels as characteristics and, as labels or classes, the math performances of individuals participating in specially designed experiments. The proposed methodology is based on using well-established procedures of feature selection, which were used to determine a suitable brain functional network size related to math problem solving strategies and also to discover the most relevant links in this network without including noisy connections or excluding significant connections.

## 1. Introduction

Recently, there has been an outburst in the number of investigations related to the applications of data mining tools to neuroscience [1, 2]. Data mining in this domain is usually related, on one hand, to processing/analyzing three-dimensional images from different medical imaging modalities that capture structural (e.g., MRI, CT, and histology) and functional/physiological (e.g., PET, fMRI, and SPECT) information about the human brain [3, 4]. On the other hand, some tools and approaches have been specifically tailored to grasp the complexity of brain electric activity through the analysis of electroencephalographic (EEG) signals [5]. However, the vast majority of these studies commonly seek to discover patterns in electrophysiological signals and images correlated with the diagnosis, prognosis, and evolution of a particular pathology or brain disorder and with the image analysis of normal/disease resting state fMRI [6, 7]. Comparatively, only very few works in this area use machine learning techniques for studying normal brain cognitive high level functions; probably because in these cases, the interpretation of the effects of single brain regions or connections between these regions on the separation of *pattern classes* is more complicated, given that discriminative brain pattern is a description of the cumulative contributions of many features that contribute to cognitive underpinning of brain high-level functions.

In this work, we use machine learning techniques to discover patterns of synchrony in functional brain networks, constructed from the EEG registers of a group of healthy individuals while they were solving specially designed math problems. The problems were devised specifically to detect and measure analytic processing. An intuitive resolution could lead to a quick and simple but incorrect response that should be overridden analytically. This study aims to correlate types of responses (correct or incorrect) with specific patterns of neural synchronization. The primary finding is that classification of these patterns using data mining tools on datasets from complex cognitive processes related to math performance is achievable. For each pair of EEG channels, corresponding to time windows associated with correct and incorrect answers given by the participants, correlations and phase synchrony were calculated. With these measures as entries, we construct connectivity (synchronization) networks as proxies of functional brain networks. A novel feature selection methodology that identifies the most relevant connections in these networks is proposed by using a nonlinear SVM-based classifier. This methodology allows us to determine not only a suitable network size but also the most relevant connections in the network, reducing the complexity and, therefore, facilitating the interpretation of mined patterns.

### 1.1. Synchronization/Correlation Networks of Normal Brain Cognitive High-Level Functions Used in Resolution of Math Problems

Lately, investigations have shifted from the study of local activation of large groups of neurons to the analysis of integration patterns among these groups. It is thought that the physiological bases of information processing and mental representation are provided by functional networks [8]. In fact, there is a great deal of current interest in the recent development of different techniques to extract large-scale functional and anatomical brain connectivity networks based on methods for creating correlation networks [9–11].

Researchers have developed a widely used method for creating correlation networks by using neural synchronization. Neural synchronization is a fundamental process in cortical computation which is believed to play an important role in information processing in the brain at both cellular and macroscopic levels [12, 13]. Brain oscillations that are ubiquitous phenomena in all brain areas become synchronized and consequently allow an implementation of the whole range of brain functions [12]. In particular, in our work, we use neural synchronization to measure the integrated activity of the functional brain network responsible for different math performances. Specifically, we use linear correlation and phase synchronization as measures for neural synchronization.

The correlation coefficient estimates linear coupling among signals of EEG channels, and its values are distributed over the unit interval. But the assumption that only linear interdependencies are relevant is actually not correct. Strictly speaking, linear correlation analysis based on Pearson's correlation coefficient and its derivatives can potentially miss important features of any dynamic system, particularly when we study brain functional network integration dynamics. Thus, in addition to linear correlation, we use phase synchronization between distant brain oscillating foci [14–18].

Phase synchrony in EEG channels assesses the stability of differences between phases of EEG signals at equivalent frequencies taken simultaneously by different electrodes. More simply stated, it is a measure of how the relative phase is distributed over the unit circle. If the two signals are phase synchronized, the relative phase will occupy a small portion of the circle, and the mean phase coherence is high. Phase synchronization has previously been considered to be a very good indicator of the functional coupling of neural activity in distant brain areas [19, 20]. To our knowledge, to date, phase synchrony in EEG channels has not been used to study brain networks involved in mathematical activities. So, using it creates a relevant contribution to understanding the collaborative and integrative nature of neural functioning in mathematics.

The large-scale functional integration of different brain zones is a relevant aspect of understanding the neural mechanisms responsible for the use of diverse problem-solving strategies in mathematics. The cognitive underpinnings of several mathematical activities have previously been related to a widely distributed brain network that includes parietal, temporal, and frontal structures as their main nodes [21–23]. Our research uses electroencephalographic (EEG) analysis [18] for the study of the whole-brain connectivity network and shows how mathematical cognition depends upon the integration of activities from distributed brain regions.

Some researches on EEG analysis have shown that specific aspects of mathematical reasoning could be related to different features of electric activity in some frequency bands (see, e.g., [24, 25]). In [25], for example, it is shown that incorrect performance in simple mathematical tasks is preceded by higher delta activity (signal frequencies < 4 Hz) in the lateral and medial areas of the right prefrontal cortex and by higher theta activity (4–8 Hz) bilaterally in the medial frontal zones. These slow wave patterns precede the subject's erroneous performance and show inhibited activity of the error-monitoring areas during erroneous mathematical calculations (i.e., these areas were simply not recruited). Therefore, a failure in the functional integration of these zones during problem resolution would be responsible for the subject's erroneous mathematical performance. On the other hand, correct answers were preceded by alpha activity (8–12 Hz) in the right posterior parietal area, a zone previously linked to mathematics. These early findings suggest that the size and integration of the functional network of different brain zones entailed in the resolution of problems are a relevant issue for understanding the neural mechanisms underlying math performance.

### 1.2. Graph Theoretical (Network) Approaches and SVM Working Together

Network theory is helpful in characterizing the interdependencies of various brain zones. However, graph theoretical (network) approaches in the study of brain functional networks suffer from some important methodological difficulties [26, 27]. For example, graph measures are strongly dependent on the network size (number of nodes), network density (percentage of links present), and degree (number of connections per vertex). This makes comparing results from different studies, which generally use distinct criteria to build functional networks, very difficult. Indeed, to construct unweighed networks, one has to apply a threshold on the connectivity values of the original weighted network. This results in scaling of the network properties as a function of the threshold [26]. The threshold can be chosen in a variety of ways, for example, based on an arbitrary choice, or using statistical criteria of connectivity strength, based on the average degree, or based on the density of the network. Fixing a standard number of vertices and the average degree could solve these size effects but could also introduce spurious connections or ignore strong connections in the network [27].

Recently, the use of a minimum spanning tree (a subnetwork of the original weighted network that connects all vertices in the network without forming loops and has the minimum total weight of all possible spanning trees), see [28], has been proposed to solve many of these methodological difficulties.

### 1.3. Top-Down Approach and Main Motivations for This Study

Students often arrive at universities without a well-formed background in abstract reasoning and with limited experience in the application of mathematical strategies. They lack proper understanding of some mathematical topics and often use inappropriate associations of different facts while trying to solve mathematical problems. These associations are fast internal reactions to external stimuli and appear to be related to the way in which the mind processes information.

Many authors in educational research have pointed out the persistence of student errors and misconceptions with respect to specific topics and tasks. For example, in [29], the authors observed that students react in a similar way to a wide variety of conceptually nonrelated problems that share some external common features. This fact led them to suggest that many responses described in the literature as alternative conceptions (misconceptions) could be better explained as evolving from a few common intuitive rules such as *More of A—More of B*, *Same A—Same B*, *Everything can be divided*, and *Over-generalized linearity*.

The present work applies a dual-process model of cognitive processing to these kinds of problems, testing the hypothesis that relative amounts of intuitive/analytic processing by the brain promote different strategies in the resolution of mathematical problems, leading to accurate or faulty solutions.

This work aims to solve these methodological difficulties by using some advanced tools from data mining. Specifically, the main methodological contribution is twofold: First, we extend the *ξ*-SOCP method [30], originally developed for linear binary classification, to nonlinear modeling thanks to the use of kernel functions. This model proposes a robust setting based on second-order cone programming, in which the traditional maximum margin approach for SVM is adapted by replacing the reduced convex hulls by ellipsoids [31], leading to a potentially superior classification performance [30, 31]. Additionally, we propose a novel feature selection methodology that identifies the most relevant connections in the network of interest while constructing the classifier using the *ξ*-SOCP method [30].

The rest of this article is structured as follows: Section 2 presents the methodology for capturing the data used in the modelling process.  Section 3 provides a brief description of developments for feature selection and SVM, in which our *ξ*-SOCP method and the novel-embedded feature selection strategy is highlighted.  Section 4 describes our results using neural synchronization datasets collected for this study. A summary of this paper can be found in Section 5, where we provide the main conclusions of this study and address future developments.

## 2. Materials and Methods: Cognitive Neuroscience

### 2.1. The Dual Process Theory

As our theoretical framework, we use the dual process theory (DPT) [32, 33]. According to DPT, our cognition and behavior operate in parallel with two quite different modes, called system 1 (S1) and system 2 (S2), roughly corresponding to our commonly held notions of intuitive and analytical thinking. The S1 and S2 modes are activated by different parts of the brain and have different evolutionary origins (S2 being more recent evolutionary and, in fact, largely reflecting cultural evolution). Like perception, S1 processes are characterized by being fast, automatic, effortless, unconscious, and inflexible (hard to change or overcome). Unlike perception, S1 processes can be language-mediated and relate to events not in the here-and-now (i.e., events in faraway locations and in the past or future). In contrast, S2 processes are slow, conscious, effortful, and relatively flexible. The two systems differ mainly along the dimension of accessibility: how fast and how easily things come to mind. Although both systems can at times run in parallel, S2 often overrides the input of S1 when analytic tendencies are activated and cognitive resources are available. For example, it is known that in geometry-related math problems, students tend to handle attributes of the problems such as distance, size, and similarity that are automatically registered by S1 quickly and spontaneously. We used this fact to design tests with some salient stimuli in such a way that each alternative for answering the problem would clearly indicate whether the participant took an intuitive/wrong strategy or an analytic/correct one.

The use of S2, consciously accessed, analytical processes trigger global and large-scale patterns of integrated neural activity. This fact appears as a variation on the global amount of synchrony between different brain areas. A greater proportion of S2 processes will appear as a greater amount of global synchrony. On the other hand, typical math errors due to semiautomatic use of heuristics will appear neurally as a reduced coupling of central work space neurons. Central work space neurons are thought to be particularly dense in the parietal, prefrontal, and cingulate cortices [21].

### 2.2. Test Designing Based on Cognitive Neuroscience

DPT enables understanding diverse phenomena because it predicts different judgments qualitatively depending on which reasoning system is used. DPT has been applied successfully to diverse domains and phenomena across a wide range of fields. While heuristic processing may render some manageable mathematics problems (by reducing the number of consciously driven operations), on some occasions, it can lead to errors and bias, reducing the effectiveness of a strategic plan of resolution. Available evidence and theory suggest that a converging suite of intuitive cognitive processes facilitates and supports some common rule-based flaw strategies in the resolution of math problems, which is a central aspect of deficient mathematical performance. In this way, stereotyped errors come from the semiautomatic and insufficiently evaluated application of highly repeated S1 system heuristics for solving problems. Under most circumstances, S1 procedures lead to correct answers (e.g., linearity is a common property of many, but not to all, mathematical operations) but in certain cases, it can lead to mistakes. To avoid these errors, the subjects must inhibit their semiautomatic responses to allow proper, conscious evaluation of the problem [34]. Some neuroscience researches have linked response inhibition to prefrontal activity, especially in its medial zones [35]; error monitoring in general (see [36], for detailed review) and mathematical error monitoring in particular [22] have been linked to the frontal lobes, mainly to their medial structures.

However, individual differences in the tendency to override initially flawed intuitions in reasoning analytically could be associated with different mathematical performances. In fact, elaborative processing must entail a deeper level of consciously controlled stimulus analysis. This processing is assumed to involve more effortful, analytical thought and is less likely to lead to errors and biases, although sometimes it may prove to be dysfunctional due to effects such as *paralysis by analysis*—the tendency to become overwhelmed by too much information processing.

Some attributes of the problem denominated in DPT as *natural assessments* could lead to wrong strategies and answers, because students could ignore other, less accessible, attributes of the problem, or some instructions that should be considered in the resolution.

Another possible source of errors is called *attribute substitution*. According to [32], when people try to solve a complex problem, they often substitute attributes. That is to say, an individual assesses a specific real attribute of the problem heuristically by means of another attribute, which comes to mind more easily. The real attribute is less accessible, and another, related attribute which is more available replaces the first one. This substitution is so fast that S2 monitoring functions cannot be activated. The individual does not notice that he/she is really answering another question.

The math tasks in our experiments were designed to highlight different problem resolution strategies. An intuitive approach, for example, will produce a quick and easy, yet incorrect, answer that must be analytically overridden to be correct. In every case, participants choosing different resolution strategies will at the same time choose different alternatives to answer the math problem. Appendix B presents three of the math problems for illustrative purposes. The complete list of the 20 math problems can be found as supplementary material (available here).

### 2.3. Preprocessing the Dataset from EEG Recording

The raw data for the training and test subsets (see the next sections) were extracted from the EEGs of a group of engineering students that were recorded while each one of them was solving a set of 20 math problems. The relevant metadata for the various participants is presented in Appendix A. These EEGs (10–10 position convention) were registered in a semidark room with a low level of environmental noise while each student was sitting in a comfortable chair. The data were recorded with the 64-channel Geodesic Sensor Net (EGI, USA) at the sampling frequency of 1000 Hz.

Since the sensors in the outer ring of the net were excluded from the analysis, because of low-quality signals, only 61 sensors were used for computations. The data were previously filtered (FIR, band-pass of 1–100 Hz), rereferenced against the common average reference, and segmented into nonoverlapping 1-s epochs using NS3 software.

As preliminary work for cleaning the dataset, we separated the oscillatory EEG-evoked electric activity from the induced one [37]. To do this, the EEG-evoked activity for each subject and his/her specific math problem was measured and averaged. This evoked activity was then subtracted from the total EEG activity through tests, subjects, and electrodes. The resulting EEG subtraction signal was analyzed with a fast Fourier transform on mobile overlapping and longtime windows between 5 and 10 seconds, because we did not know a priori what the interesting cognitive events to measure would be.

The measurement for each subject-math problem was segmented into time intervals ranging from −0.1 s to 61 s. In *t* = 0, the math problem is presented, while at *t* = 60 s, the question mark appears. The value −500 ms is considered to be the baseline of before the occurrence of the problem.

### 2.4. Constructing the Correlation and Synchronization Matrices from the Raw Dataset

As will be shown in the following sections, a new method for feature extraction from EEG signals was developed by choosing elements of the correlation or synchronization matrices. The EEG time series recorded for each participant/math problem were used to construct the correlation and synchronization matrices of the functional brain networks with rows and columns representing sensors. These matrices contain information about (linear) interdependence and long-range synchronies between EEG channels. Both types of information would be used for classification purposes. Moreover, in the case of the synchronization matrix, we would also manage information about frequency bands.

The correlation coefficient *r*_*x*,*y*_ is perhaps one of the most well-known measures for (linear) interdependence between two signals *x* and *y*:

(1) $$\begin{array}{c} r_{x,y}=\frac{1}{N}\overset{N}{\underset{k=1}{\sum }}\frac{\left(x\left(k\right)-\bar{x}\right)\left(y\left(k\right)-\bar{y}\right)}{\sigma_{x}\sigma_{y}}, \end{array}$$

where *N* is the length of the signals, $\bar{x}$ and $\bar{y}$ are the (sample) means of *x* and *y*, respectively, and *σ*_*x*_^2^ and *σ*_*y*_^2^ are the (sample) variances of *x* and *y*, respectively.

The correlation coefficient *r*_*x*,*y*_ quantifies the linear correlation between *x* and *y*. If *x* and *y* are not linearly correlated, *r*_*x*,*y*_ is close to zero; on the other hand, if both signals are identical, then *r*_*x*,*y*_ = 1.

Every correlation coefficient *r*_*i*,*j*_ is a bivariate measure that serves as a coupling coefficient that links the electrode nodes *i* and *j*. With these coefficients as entries, we construct a connectivity matrix (adjacency matrix) **Corr**, representing a functional brain network. Thus, we have a connectivity matrix 61 × 61 composed of undirected and weighted edges consistent with the correlation coefficients. The matrix **C** **o** **r** **r** is symmetric, so it has *N*(*N* − 1)/2 = 1830 independent elements. Zeros are placed in diagonal elements.

In order to discover to what extent two sensor locations were synchronized, we also used the phase locking value (PLV) [18, 19]. Sample PLV is one of the most widely used measures of brain synchronization. It quantifies the phase relationship between two signals with high temporal resolution without making any statistical assumptions on the data.

Given two time series of signals *x*(*t*) and *y*(*t*) and a frequency of interest *f*, the procedure computes a measure of phase locking between the components of *x*(*t*) and *y*(*t*) for each latency at frequency *f*. This requires the extraction of the instantaneous phase of every signal at the target frequency. The phases are calculated by convolving each signal with a complex wavelet function:

(2) $$\begin{array}{c} \Psi \left(t\right)=e^{-\left(t^{2}/2{\sigma_{t}}^{2}\right)}e^{2i\pi ft}, \end{array}$$

that is,

(3) $$\begin{array}{c} W_{x}\left(t\right)={\left(\Psi \circ x\right)}_{t}=\int \Psi \left(t^{\prime }\right)x\left(t^{\prime }-t\right)dt^{\prime }=A_{x}^{W}\left(t\right)e^{i\Phi_{x}^{W}\left(t\right)}, \end{array}$$

where *A*_*x*_^*W*^(*t*) represents the signal amplitude. Following [19], we take *σ*_*t*_ = 7/*f* and we define *W*_*y*_(*t*) in the same way as *y*(*t*). Next, we can calculate the phase differences *Φ*_*x*,*y*_^*W*^(*t*) = *Φ*_*x*_^*W*^(*t*) − *Φ*_*y*_^*W*^(*t*). The phase locking value is then defined at time *t*, as the average value:

(4) $$\begin{array}{c} {PLV}_{x,y}\left(t\right)=\frac{1}{N}\;\left|\overset{N}{\underset{n=1}{\sum }}e^{{i\Phi }_{x,y}^{W}\left(t,n\right)}\right|, \end{array}$$

for all time-bins *t* and trial *n* ∈ {1,…, *N*}.

In our experiments, PLV measures were normalized relative to a baseline [38]. Specifically, this was done by using the 500 ms baseline before the onset of the math problem. The normalized signal was obtained by subtracting the average activity of the baseline from the raw signal and then dividing by the standard deviation of the baseline in a frequency-by-frequency manner.

By construction, PLV will be zero if the phases are not synchronized at all and will be one when the phase difference is in perfect, constant synchronization. The key feature of PLV is that it is only sensitive to phases, irrespective of the amplitude of each signal.

From the *N* = 61 EEG channels, we computed a symmetric 61 × 61 synchronization matrix **S** for each participant and for each math problem within a specific frequency band. Each element **S**_*i*,*j*_ of the matrix **S** corresponds to the PLV computed for the electrode pair *i* and *j*PLV_*i*,*j*_(*t*). The matrix **S** is also symmetric, so it has *N*(*N* − 1)/2 = 1830 independent elements and, as before, zeros are placed in diagonal elements.

**Figure 1:**
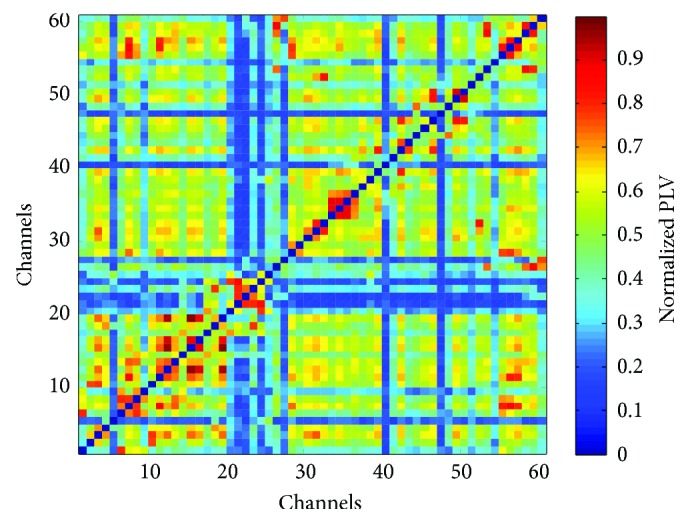
An example of the synchronization matrix for an individual and specific mathematics problem, in this case, corresponding to the beta (14–30 Hz) band. The colors indicate the extent to which two sensor locations are synchronized, which are quantified by the normalized PLV between every two EEG-recording sites *i* and *j* with *i*, *j* = 1,…, 61.

Each matrix element of **S** is the PLV computed for the corresponding pair of sensors. An illustrative example of the synchronization matrix is presented in Figure 1.

### 2.5. From the Correlation (**C** **o** **r** **r**) and Synchronization (**S**) Matrices to a Binary Classification Problem

For classification purposes, we prepared two datasets using the correlation **C** **o** **r** **r** and synchronization **S** matrices separately. Each data point corresponds to an answer of a participant to a specific mathematics problem. The participant could answer the math problem in a correct way (*y* = +1) or incorrectly (*y* = −1).

With the help of these matrices, we further constructed feature vectors **x** of size *N*(*N* − 1)/2, whose components are in one case the elements of the matrix **C** **o** **r** **r**, and in other cases elements of the matrix **S**. Here, *N* is the number of EEG sensors used in signal recordings, and we also used the fact that both matrices **C** **o** **r** **r** and **S** are symmetric.

This way, the datasets for the learning machine are given by the set:

(5) $$\begin{array}{c} \left(x_{1}^{\left(a\right)},y_{1}\right),\ldots ,\left(x_{l}^{\left(a\right)},y_{l}\right), \end{array}$$

with characteristic vectors **x** ^(*a*)^ ∈ *ℜ* ^*N*(*N* − 1)/2^, *a* = {**C** **o** **r** **r**, **S**} corresponding to **C** **o** **r** **r** or **S** matrix entries, respectively, and the output label *y*_*l*_ ∈ {−1, +1}, where the subindex *l* denotes the number of participants multiplied by the number of math problems given to each participant.

Thus, with the previously preprocessed dataset collected from 14 participants, with a 61-channel (*N* = 61) EEG Geodesic Sensor Net (EGI) and with 20 math problems for each of them, we have *l* = 14 × 20 = 280 sample vectors of dimension *N*(*N* − 1)/2 = 1830 to train SVM models and make a preliminary classification for each **C** **o** **r** **r** and **S** cases.

For the case of synchronization matrix **S**, we further developed the datasets considering the analysis for frequencies in three distinct domains, which we have called as follows: *Low*, corresponding to *δ* (<4 Hz) and *θ* (4–8 Hz) bands; *Medium*, corresponding to the *α* (8–12 Hz) and *β* (14–30 Hz) bands; and *High*, corresponding to *γ* (30–80 Hz) bands. In the case of the *Medium* frequency domain, we also studied the *β* band in a separate way. A classification study is performed using each frequency domain for synchrony detection in order to find the one that lead to the best classification.

## 3. Materials and Methods: Support Vector Machines and Feature Selection

Among the existing machine learning methods, SVM has demonstrated superior performance in several domains and, in particular, in neuroscience [39]. Its appealing characteristics, such as the absence of local minima and an adequate generalization of new samples, thanks to the *structural risk minimization* principle [40], made SVM one of the preferred classification approaches among researchers and practitioners [41].

Feature selection is a very important topic in high-dimensional applications, and, in particular, in neurology [1]. Finding the adequate subset of relevant variables for a given data mining task reduces the risk of overfitting, improving the model's predictive performance, and provides important insight into the process that generates the data, enhancing the interpretability of the model [42]. Support Vector Machine, however, cannot derive the feature's importance within the respective model, and therefore variable selection methods need to be used in order to reduce the level of noise in high-dimensional datasets [43].

In this section, the traditional SVM model developed for binary classification by [44] is presented in both linear- and kernel-based versions. Subsequently, two recently developed extensions are discussed, namely, the twin SVM method [45, 46] and the SVM method based on second-order cone programming presented in [30] (*ξ*-SOCP). Finally, two feature selection approaches used to address the issue of high dimensionality are described: the Fisher score and RFE-SVM method.

Among all SVM variations, we chose Twin SVM and SOCP SVM due to their superior performance we observed in previous studies (see e.g., [30, 47]). Twin SVM has shown positive empirical performance compared with the standard SVM formulation, being also computationally more efficient since the construction of these classifiers can be done by splitting the optimization problem into two smaller subproblems [45, 46]. Regarding *ξ*-SOCP, it is based on robust optimization, considering the worst-case setting for the class conditional densities related to the two training patterns in binary classification. In machine learning, robustness is a valuable property since it reduces the risk of overfitting, guaranteeing that the test performance does not deteriorate too much compared to the training performance when slight changes in the data distribution occur [30, 47].

### 3.1. Soft-Margin SVM

Let {(**x**_*i*_, *y*_*i*_)}_*i*=1_^*m*^ be a set of examples **x**_*i*_ ∈ *ℜ* ^*n*^ with labels *y*_*i*_ ∈ {−1, +1}, *i* = 1,…, *m*. The traditional soft-margin SVM formulation [44] finds a classifier of the form **w** ^*T*^ **x** + **b** = 0 by solving the following model:

(6) $$\begin{array}{c} \underset{w,b,\xi }{\min}\;\frac{1}{2}{\left\|w\right\|}^{2}+C\overset{m}{\underset{i=1}{\sum }}\xi_{i}\\ s.t.\;y_{i}\left(w^{T}x_{i}+b\right)\geq 1-\xi_{i},\\ \;\xi_{i}\geq 0,\\ \;i=1,\ldots ,m. \end{array}$$

For each training example, a slack variable *ξ*_*i*_ is introduced, while *C* is a positive parameter that controls the trade-off between margin maximization and model fit.

A nonlinear decision surface can be obtained by using a kernel function [48]. A maximum margin classifier is constructed in a higher dimensional space by computing the dual of Formulation 4 and applying the kernel trick, leading to the following problem:

(7) $$\begin{array}{c} \underset{\alpha }{\min}\;\frac{1}{2}\overset{m}{\underset{i,s=1}{\sum }}\alpha_{i}\alpha_{s}y_{i}y_{s}K\left(x_{i},x_{s}\right)-\overset{m}{\underset{i=1}{\sum }}\alpha_{i}\\ s.t.\;\overset{m}{\underset{i=1}{\sum }}\alpha_{i}y_{i}=0,\;0\leq \alpha_{i}\leq C,\;i=1,\ldots ,m, \end{array}$$

where **α** is a vector in *ℜ* ^*m*^ of the dual variables corresponding to the constraints in 4 and *K* : *ℜ* ^*n*^ × *ℜ* ^*n*^ → *ℜ* is a kernel function. A typical choice of kernel is the *Gaussian kernel*, which usually leads to better results [43]. This kernel is as follows:

(8) $$\begin{array}{c} K\left(x_{i},x_{s}\right)=\exp\left(-\frac{{\left\|x_{i}-x_{s}\right\|}^{2}}{2\sigma^{2}}\right), \end{array}$$

where *σ* > 0 is the kernel width parameter [46].

### 3.2. Twin Support Vector Machine

The twin SVM method [45] constructs two nonparallel hyperplanes instead of the single classifier used in the soft-margin SVM formulation. Formally, two hyperplanes of the form **w**_1_^*T*^ **x** + **b**_1_ = 0, **w**_2_^*T*^ **x** + **b**_2_ = 0 are obtained in such a way that each of the functions is closer to the samples of one of the two labels and, at the same time, is as far as possible from those points of the other class. The following two problems are solved in order to find the following hyperplanes:

(9) $$\begin{array}{c} \begin{array}{c} \underset{w_{1},b_{1},\xi_{2}}{\min}\;\frac{1}{2}{\left\|Aw_{1}+e_{1}b_{1}\right\|}^{2}+\frac{c_{3}}{2}\left({\left\|w_{1}\right\|}^{2}+b_{1}^{2}\right)+c_{1}e_{2}^{T}\xi_{2}\\ s.t.\;-\left(Bw_{1}+e_{2}b_{1}\right)\geq e_{2}-\xi_{2},\;\xi_{2}\geq 0, \end{array}\\ \begin{array}{c} \underset{w_{2},b_{2},\xi_{1}}{\min}\;\frac{1}{2}{\left\|Bw_{2}+e_{2}b_{2}\right\|}^{2}+\frac{c_{4}}{2}\left({\left\|w_{2}\right\|}^{2}+b_{2}^{2}\right)+c_{2}e_{1}^{T}\xi_{1}\\ s.t.\;\left(Aw_{2}+e_{1}b_{2}\right)\geq e_{1}-\xi_{1},\;\xi_{1}\geq 0, \end{array} \end{array}$$

where **A** and **B** are the data matrix for the positive and negative class, respectively, *c*_*i*_ are trade-off positive parameters (*i* = {1, 2, 3, 4}), and **e**_1_ and **e**_2_ are vectors of one's appropriate dimension. Previous formulation is known as twin-bounded SVM (TB-SVM) [46], which is similar compared to the twin SVM (TW-SVM) method proposed by Jayadeva et al. [45] when setting *c*_1_ = *c*_2_ = *ε*.

Similarly to the soft-margin formulation, a nonlinear decision surface can be obtained by applying the kernel trick. The kernel-based twin SVM method solves the following two problems:

(10) $$\begin{array}{c} \begin{array}{c} \underset{u_{1},b_{1},\xi_{2}}{\min}\;\frac{1}{2}{\left\|K\left(A^{T},X\right)u_{1}+e_{1}b_{1}\right\|}^{2}+\frac{c_{3}}{2}\left({\left\|u_{1}\right\|}^{2}+b_{1}^{2}\right)+c_{1}e_{2}^{T}\xi_{2}\\ s.t.\;-\left(K\left(B^{T},X\right)u_{1}+e_{2}b_{1}\right)\geq e_{2}-\xi_{2},\;\xi_{2}\geq 0, \end{array} \end{array}$$

(11) $$\begin{array}{c} \begin{array}{c} \underset{u_{2},b_{2},\xi_{1}}{\min}\;\frac{1}{2}{\left\|K\left(B^{T},X\right)u_{2}+e_{2}b_{2}\right\|}^{2}+\frac{c_{4}}{2}\left({\left\|u_{2}\right\|}^{2}+b_{2}^{2}\right)+c_{2}e_{1}^{T}\xi_{1}\\ s.t.\;\left(K\left(A^{T},X\right)u_{2}+e_{1}b_{2}\right)\geq e_{1}-\xi_{1},\;\xi_{1}\geq 0, \end{array} \end{array}$$

where *𝕏* = [**A** ^*T*^ **B** ^*T*^] is the matrix of both training patterns (sorted by class) and *K* : *ℜ* ^*n*^ × *ℜ* ^*n*^ → *ℜ* is the kernel function.

Finally, a new data point is assigned to label +1 (*k* = 1) or −1 (*k* = 2) according to its proximity to the two hyperplanes. That is, **x** ∈ *ℜ* ^*n*^ belongs to the label *k* ^∗^ iff

(12) $$\begin{array}{c} k^{*}=\underset{k=1,2}{argmin}\left\{\frac{\;|\;w_{k}^{T}x+b_{k}\;|\;}{\left\|w_{k}\right\|}\right\}. \end{array}$$

The twin SVM method has been recently applied in neuroscience [49] and, in particular, in pattern analysis with EEG signal data [50, 51].

### 3.3. *ξ*-Second-Order Cone Programming SVM

In this study, we also used the robust SVM version based on second-order cones presented by [30]. For instance, if we suppose that **X**_1_ and **X**_2_ are random vector variables that generate samples of positive class (brain synchrony pattern of a participant that made a good resolution of the math problem) and negative class (brain synchrony pattern of participant that made an incorrect resolution of the math problem), respectively, we should construct a maximum margin linear classifier such that the false-negative and false-positive error rates do not exceed *η*_1_ ∈ (0, 1] and *η*_2_ ∈ (0, 1], respectively, in the following Quadratic Chance-Constrained Programming (QCCP) problem:

(13) $$\begin{array}{c} \underset{w,b,\xi }{\min}\;\frac{1}{2}{\left\|w\right\|}^{2}+C\xi \\ s.t.\;\mathrm{Pr}\left\{w^{T}X_{1}+b\geq 1-\xi \right\}\geq \eta_{1},\\ \;\mathrm{Pr}\left\{w^{T}X_{2}+b\leq -1+\xi \right\}\geq \eta_{2},\\ \;\xi \geq 0. \end{array}$$

False negative could appear, for example, due to not completely reliable math assessment. In this case, there is a possibility of correct answers to a math problem, despite that it is registered as a pattern of low synchronization in the brain activity, that is, when a student correctly solves the problem without performing a deep analytical thinking.

The proposed robust setting suggests classifying each label correctly, up to the rate *η*_*k*_, even for the worst data distribution. Thanks to Chebyshev's inequality [52, Lemma 1], this approach leads to the following deterministic problem:

(14) $$\begin{array}{c} \underset{w,b,\xi }{\min}\;\frac{1}{2}{\left\|w\right\|}^{2}+C\xi \\ s.t.\;w^{T}\mu_{1}+b\geq 1-\xi +\kappa_{1}\sqrt{w^{T}\Sigma_{1}w},\\ \;-\left(w^{T}\mu_{2}+b\right)\geq 1-\xi +\kappa_{2}\sqrt{w^{T}\Sigma_{2}w},\;\xi \geq 0, \end{array}$$

where **μ**_*k*_ and *Σ*_*k*_ are the means and covariance matrices for each class *k* = 1, 2 and $\kappa_{k}=\sqrt{\eta_{k}/1-\eta_{k}}$, and *C* > 0 is a tradeoff parameter. The constraints appearing in the previous problem are called second-order cone constraints [53]. Thus, we refer to this problem as the *ξ*-SOCP formulation.

Similar to [54], a nonlinear version can be also derived for *ξ*-SOCP via the kernel trick. The kernel-based *ξ*-SOCP method is as follows:

(15) $$\begin{array}{c} \underset{s,b,\xi }{\min}\;\frac{1}{2}s^{T}Ks+C\xi \\ s.t.\;s^{T}g_{1}+b\geq 1-\xi +\kappa_{1}\sqrt{s^{T}\Xi_{1}s},\\ \;-\left(s^{T}g_{2}+b\right)\geq 1-\xi +\kappa_{2}\sqrt{s^{T}\Xi_{2}s},\;\xi \geq 0, \end{array}$$

where **K** = [**K**_11_, **K**_12_; **K**_21_, **K**_22_] ∈ *ℜ* ^*m*×*m*^, with **K**_11_ = *AA* ^*T*^, **K**_12_ = **K**_21_^*T*^ = *BA* ^*T*^, **K**_22_ = *BB* ^*T*^ and

(16) $$\begin{array}{c} g_{k}=\frac{1}{m_{k}}\left[\begin{array}{c} K_{1k}e_{k}\\ K_{2k}e_{k} \end{array}\right],\\ \Xi_{k}=\frac{1}{m_{k}}\left[\begin{array}{c} K_{1k}\\ K_{2k} \end{array}\right]\left(I_{m_{k}}-\left(\frac{1}{m_{k}}\right)e_{k}e_{k}^{T}\right)\left[K_{1k}^{T}\;K_{2k}^{T}\right]. \end{array}$$

### 3.4. Feature Selection for SVM

In this work, two feature selection strategies that have been used frequently for binary classification with SVM are considered: the Fisher score and RFE-SVM [42]. The first technique assesses variable relevance before applying SVM, constructing a ranking of attributes that can be used as input for the SVM model. This ranking is based on the distance between *μ*_*j*_^+^ and *μ*_*j*_^−^, the means for the *j*th attribute in the positive and negative labels are as follows:

(17) $$\begin{array}{c} F\left(j\right)=\left|\frac{\mu_{j}^{+}-\mu_{j}^{-}}{{\left(\sigma_{j}^{+}\right)}^{2}+{\left(\sigma_{j}^{-}\right)}^{2}}\right|, \end{array}$$

where *σ*_*j*_^+^ (*σ*_*j*_^−^) is the standard deviation for the positive (negative) label. RFE-SVM, in contrast, performs a feature selection process embedded in the model, eliminating those variables that have the lowest contribution iteratively [42]. The variable contribution (the SVM margin when removing a given attribute) can be written in terms of the dual variables as follows:

(18) $$\begin{array}{c} W^{2}\left(\alpha \right)=\overset{N}{\underset{i,s=1}{\sum }}\alpha_{i}\alpha_{s}y_{i}y_{s}K\left(x_{i},x_{s}\right). \end{array}$$

The RFE-SVM algorithm was successfully applied to linear Twin SVM in [55]. However, the RFE algorithm has not been extended before to *ξ*-SOCP, to the best of our knowledge. In this work, we implement the RFE algorithm for the *ξ*-SOCP method. This strategy together with the kernel-based *ξ*-SOCP formulation is a novel methodological contribution of this work. We should note that the characteristics of feature vectors **x** are elements of the synchronization matrices, which represent the weights of network edges. So, this procedure determines the most significant features or, in other words, the most noticeable network edges that make the difference in separation of the strategies used in the resolution of mathematical problems, in a correct or incorrect way.

## 4. Results and Discussion

We applied the classification and feature selection approaches described in the previous section on five different datasets corresponding to correlation data **C** **o** **r** **r** and to synchronization data **S** which comprise three frequency domains (*Low*, *Medium*, and *High*) and the *β* frequency band. Each of the datasets has 280 samples (100 right answers and 180 wrong answers) described by 1830 variables.

For model evaluation, we chose a nested cross-validation (CV) strategy: training and test subsets were obtained using a 10-fold CV (outer loop), and the training subset was further split in training and validation subsets in order to find the right hyperparameter setting. The final feature ranking and classification were then performed with the full training subset from the outer loop for the best combination of hyperparameters, and the classification performance was computed by averaging the test results. This way, the test subsets from the outer loop remain unseen during the hyperparameter selection procedure. The following values for the hyperparameters were studied: *C*,  *c*_*i*_ and *σ* ∈ {2^−7^,…, 2^7^} and *η*_*k*_ ∈ {0.2, 0.4, 0.6, 0.8}.

All experiments were performed on an HP Envy dv6 with 16 GB RAM, 750 GB SSD, an Intel Core Processor i7-2620 M (2.70 GHz), and using Microsoft Windows 8.1 OS (64 bits). The toolbox LibSVM [56] was used for standard SVM approaches, while the SeDuMi Matlab Toolbox [57] and the codes provided by Shao et al., the author of Twin-Bounded SVM [46] (publicly available in http://www.optimal-group.org/), were used for *ξ*-SOCP and TB-SVM, respectively.

Table 1 presents the best performance of the model selection procedure for all classification methods (standard SVM, TB-SVM, and *ξ*-SOCP in their linear and nonlinear versions) and for all five datasets without performing feature selection. The average performance among all techniques for each data is also reported. The two best performances among all methods are highlighted in bold type.

From Table 1, we observe that *Medium* and **Corr** are the ones with better average performance among the five different datasets. For the remaining analysis, we focus on these two datasets.

Next, we studied both feature selection approaches (Fisher score and recursive feature elimination) with the following number of selected attributes: 10, 20, 50, 100, 250, 500, 1000, and 1830 (i.e., with no features removed). The nested cross-validation strategy was performed for each subset of features, and the best performance is reported in Table 2, indicating the optimal number of selected variables for each case. The best performance among all methods is highlighted in bold type for each dataset.

In Table 2, we first observe that feature selection can improve predictive performance compared to a case with all available information, confirming what the specialized literature on this topic suggests [42]. In our case, an improvement of around 2% is achieved by eliminating those attributes that introduce noise in the modelling process. The best strategies are *ξ*-SOCP in its kernel-based version in combination with Fisher score and standard linear SVM in combination with RFE for the **Corr** and *Medium* datasets, respectively. Notice that the best approach is the one with fewer selected attributes in case of ties in accuracy.

**Figure 2:**
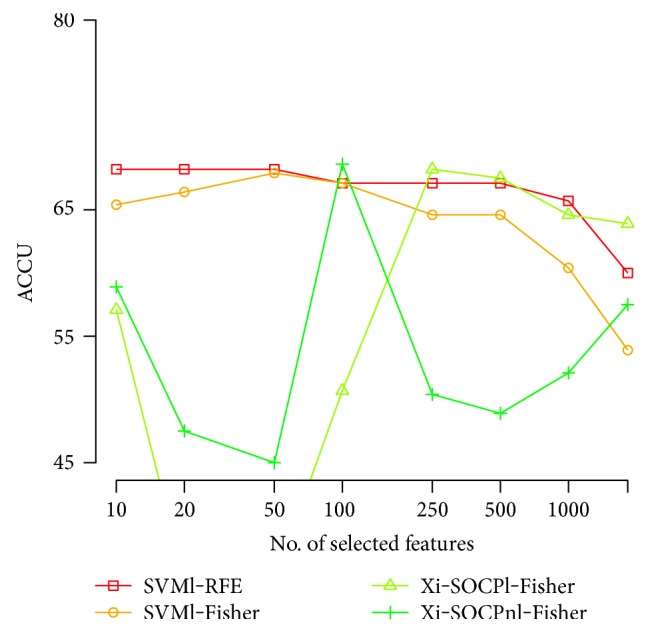
Accuracy versus the number of selected variables for **Corr** dataset.

**Figure 3:**
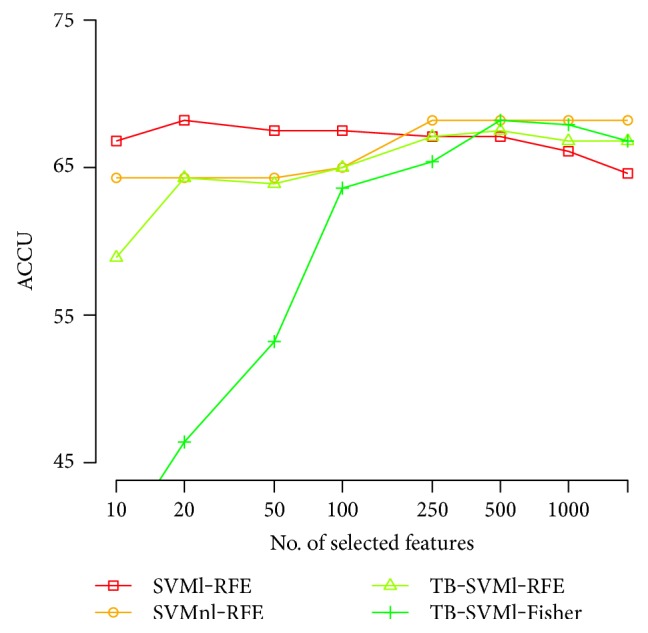
Accuracy versus the number of selected variables for *Medium* dataset.

Next, we construct the accuracy curves for the different subsets of selected variables, based on the four best methods presented in Table 2. For both frequency domains, we plot the classification performance of the four selected strategies to assess stability and overall predictive power. These results are presented in Figures 2 and 3.

In Figures 2 and 3, we first observe that no method outperformed others, and a reasonably broad range of classifiers is needed to define a good classifier adequately. The overall best performance is achieved with *ξ*-SOCP as the classification method and using Fisher score for feature ranking, although the most stable strategy corresponds to standard SVM with linear kernel, and the RFE algorithm for variable elimination. Notice also that the best performance is usually achieved between 10 and 100 attributes, discarding more than 90% of the available information.

Finally, we select the most relevant variables for each dataset. This is performed by combining the two best ranking strategies, namely, Fisher score and standard linear SVM in combination with RFE, and selecting the common variables (notice that each of these variables means a link between two EEG channels) that appear in both rankings (those with the highest importance).

**Figure 4:**
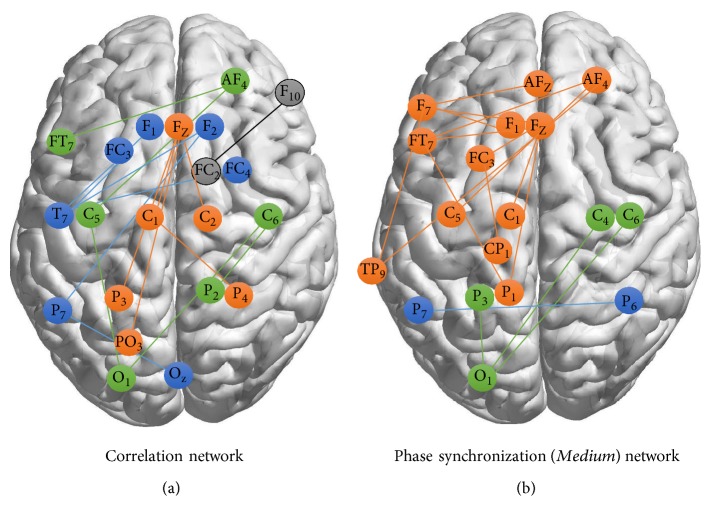
Graphic representation in which the vertices indicate EEG sensor locations (according to 10–10 position convention), and the edges represent the most significant features in the SVM classification and feature selection procedure. The colors represent different disconnected network components. BrainNet Viewer software [75] was used for the graph visualization.

Figures 4(a) and 4(b) show resulting networks constructed with statistically significant connections for the best classification methods and the set of connections that best discriminates the two classes of correct and incorrect answers during solving of math problems for correlation **C** **o** **r** **r** and synchronization **S** matrices, respectively.

## 5. Conclusions

The discriminative brain pattern is a description of the cumulative contributions of many features. Therefore, the interpretation of the effects of single brain regions or connections between regions on the separation of the pattern classes is a complicated matter. However, some marked contributions aimed to solve this problem are present in this study.

It is well known that neural synchrony, which is involved in the large-scale transient integration of functional areas widely distributed over the brain, is required for normal cognitive operations [18].  Figure 4(a) shows precisely how the correlation can and should be used to measure this integration of different functional areas in normal processes related with math problem resolution.

Despite the apparent differences in patterns of connections (that were obtained by different methodologies), there are nodes that are common to both figures: AF4, F1, Fz, FT7, CF3, C1, C5, C6, P3, P7, and O1. These nodes precisely correspond to a widely distributed brain network previously identified as related to several mathematical activities [21–23].

The most discriminative connections (selected features) for the **S** case happen in the *Medium* range of frequencies from 8 to 30 Hz. Interestingly, these relevant connections are present in both the *α* and *β* bands. The *α* frequency band is widely associated with attention processes [58–62], and there is a large consensus that under conditions of inattention, some stimuli fail to be seen because the subject's attention is occupied with a different task and/or with another salient stimulus [63–65]. In such cases, perceptual, lexical, and/or semantic processing of the math problems could occur in the absence of conscious perception. As a result, stronger nonconscious effects are observed that may lead to erroneous mathematical performance.

In [66], it was suggested that focused attention elicits *α* large phase locking during the processing of a target stimulus. This phase response can be interpreted as reflecting temporal attention. Thus, the *α* phase should also play a crucial role in the attentional blink phenomenon, which represents reduced ability to report a second target after identifying the first target in visual stimuli. The explanations of the phenomenon proposed so far have focused primarily on some cognitive aspects, such as attentional filters, capacity limitation, and retrieval failure processes [67, 68].

**Figure 5:**
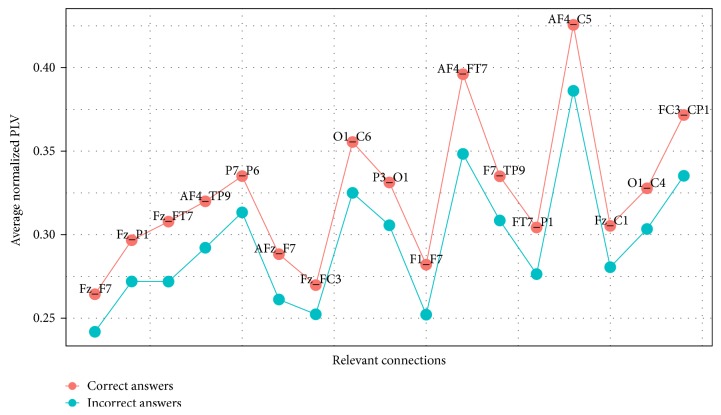
Average synchrony for relevant connections in the case of *Medium* dataset. Note that for every relevant connection, the average PLV is higher for correct answers than for incorrect ones.

Figure 5 shows the average PLV calculated for relevant connections that were determined by the procedure of feature selection explained in Section 3.4. This average was taken for all measurements made for both classes of mathematical problems, that is, the ones that were answered properly and the ones that were answered incorrectly.

Figure 5 depicts two important results. First, the measures of the synchronization in the relevant connections indicate two types of different behaviors in regard to the cases of correct responses and incorrect responses. In the first case, synchronization is consistently higher than in the case of incorrect responses, indicating a different kind of resolution strategy whose neurological correlate shows a coordinated integration of different brain areas.

Secondly, synchronization measured by PLV values is not excessively high. The interpretation of this fact is interesting. It indicates that by building a proxy of a functional brain network, using synchronization networks able for distinguishing underlying normal cognitive processes in solving complex problems, the crucial point is not the detection of a high level of synchronization but rather the determination of which areas or groups are integrated with a stable synchronization. Traditional methods based on the choice of a threshold value in correlation/synchronization networks (e.g., using a threshold value greater than 0.5) would exclude much of the analysis described in this paper, which inevitably cause the loss of relevant information related to the neurological correlates that support the cognitive processes studied.

The primary finding of this study is that classification using automated SVM of datasets from complex cognitive processes related to math performance is feasible. Moreover, feature selection is a valuable procedure for reducing the complexity and, therefore, facilitating the interpretation of the mined patterns.

The observed differences in the math performance of participants and the success of SVM classification with a reasonable statistical significance suggest the potential use of this methodology as a novel approach to study patterns of connectivity in functional brain networks related to normal cognitive processes beneath the execution of complex tasks.

A still unsolved problem in graph theoretical approach for studying brain functional network integration is that network threshold can be chosen in a variety of ways based on an arbitrary choice or using statistical criteria of connectivity strength, based on the average degree or based on the network density. Particularly, when the threshold is based on a fixed number of connections in the network, this choice may result in either inclusion of spurious or noisy connections in networks (for too high density values or too high average degree) or the exclusion of relevant connections in networks (for too low density values or too low average degree) [27]. The procedure proposed here, based on feature selection tools, allows to determine not only a suitable network size by decreasing the size of feature vector but also the most relevant connections in network, that is, the ones that contribute most to the classification. As was shown in Table 2, feature selection improves predictive performance, which means that the optimal choices of the numbers of significant variables (connections) happen without including noisy connections or exclusion of significant connections.

There are some limitations to our study. First, the present analysis was performed on EEG data in sensor space, which contains some inherent spurious correlations because volume conduction causes the signal at each sensor to be a mixture of blurred activity from different inner cortical sources. More accurate inferences about anatomical locations need a source reconstruction of the activity in the cortex [69]. Second, in pattern classification, there are always uncertainties, for example, training datasets may contain incomplete information, there is input noise, there is noise in measurements, or underlying process is stochastic. As a result of such a probabilistic setting, uncertainties arise in learning from data. In order to get more reliable and reproducible results, we constructed a classifier whose misclassification rate does not exceed a defined maximum tolerable limit. For this, we presented in Section 3.3 a methodology for classification with uncertainties using SVM. However, general approaches of chance-constrained problems require the use of more suitable numerical methods for solving problems with linear constraints of probability to get computationally tractable approximations.

## Figures and Tables

**Table 1:** Performance summary for different classification approaches. All datasets.

	*High*	*Medium*	*Low*	**Corr**	Beta
SVM_*l*_	64.6	68.6	67.9	67.9	65.0
SVM_*nl*_	67.5	67.9	67.9	66.1	65.0
TB-SVM	66.1	66.8	67.5	66.4	64.6
TB-SVM_*nl*_	65.0	66.5	65.7	67.1	61.8
*ξ*-SOCP	58.6	63.2	63.9	65.7	65.3
*ξ*-SOCP_*nl*_	65.7	67.1	64.3	66.1	63.6
Average	64.6	**66.7**	66.2	**66.5**	64.2

**Table 2:** Performance summary for different feature selection approaches. *Medium* and **Corr** datasets.

	*Medium*	*n*	**Corr**	*n*
Fisher + SVM_*l*_	66.8	50	67.9	50
Fisher + SVM_*nl*_	67.1	1000	67.5	50
Fisher + TB-SVM	68.2	500	67.1	50
Fisher + TB-SVM_*nl*_	67.5	1000	67.9	500
Fisher+*ξ*-SOCP	67.1	250	68.2	250
Fisher+*ξ*-SOCP_*nl*_	66.4	500	**68.6**	100

RFE + SVM_*l*_	**68.2**	20	68.2	10
RFE + SVM_*nl*_	68.2	250	67.5	20
RFE + TB-SVM	67.5	500	67.1	20
RFE+*ξ*-SOCP	65.4	1000	68.2	10

**Table 3:** Metadata for all participants.

Subject	Gender	Age	Correct answers	% C.A	Incorrect answers	% I.A	RT (ms)
1	Male	21	7	35	13	65	Null
2	Male	22	4	20	16	80	Null
3	Female	20	5	25	15	75	Null
—	—	—	—	—	—	—	—
4	Male	20	9	45	11	55	33091.80
5	Male	20	9	45	11	55	50645.45
6	Male	21	6	30	14	70	38395.85
7	Male	24	10	50	10	50	46157.05
8	Female	21	8	40	12	60	36193.85
9	Male	19	4	20	16	80	43862.60
10	Male	22	4	20	16	80	40500.10
11	Male	20	6	30	14	70	36475.95
12	Female	20	4	20	16	80	32184.35
13	Male	23	6	30	14	70	37211.10
14	Male	23	7	35	13	65	39002.50
15	Male	22	6	30	14	70	36721.80
16	Male	20	15	75	5	25	42437.95
17	Female	23	6	30	14	70	32250.65
Ave.	—	21.24	7	36	13	64	38938.00

## Appendix

### A. Basic and Behavioral Data

The subjects in the experiment were first-year engineering students enrolled in calculus and algebra courses. All participants were right-handed, healthy individuals, without any known neurological disorder, and with an average age of 21.24 ± 1.43. The basic metadata is presented in Table 3.

### B. Mathematical Problems

Example 1 (attribute substitution). According to Kahneman et al. [32], attribute substitution often occurs when people try to solve a complex problem. This happens when an individual heuristically assesses a specific real attribute of the problem by means of another attribute, which comes easier to the mind. The real attribute is less accessible, and another related attribute, which is more available, replaces the first one. This substitution occurs so fast thatthe S2 monitoring functions cannot be activated, so it happens in a practically unconscious way. In geometry problem resolution, for example, students quickly handle attributes such as distance, dimensions, and similarity that are automatically/unconsciously recorded. These attributes are called “natural assessments.” Managing these estimators can lead to errors, essentially because students ignore less accessible attributes that are critical to successfully solve the problem. We used this fact in Problem 1. In the resolution of this problem, some students ignored the values of angles and seem to have in mind the idea that the square has twice the area of the triangle. The average score obtained by students in this problem was 1.26 of a maximum of 4, showing that this was a fairly common error.

Problem 1. In the figure, the area of the triangle BCE is *S*. The area of the square ABCD is as follows:

- 2*S*.
- $2\sqrt{3}S$. ✓

Example 2 (intuitive rules). In [29, 70, 71], the authors observed that students react in a similar way to a wide variety of conceptually nonrelated problems but share some common features. This fact allowed them to suggest that many misconceptions described by educational research literature could be explained as consequences of some few common intuitive rules, such as “More of A—More of B,” “Same A—Same B,” “Everything can be divided,” and “Over-generalized linearity.” The fundamental contribution of the intuitive rules is the observation that human response is often determined by irrelevant externalities of the tasks and not by the really important concepts and ideas. Intuitive rules showed that subjects during the resolution of a problem, under certain circumstances, can completely ignore some important data to save the immediacy. Using this fact, Problem 3 and Problem 4 were constructed. Problem 3 is intuitive and evident; the score obtained in this problem was the maximum possible: 4.0. However, equal areas do not necessarily imply equal volumes, and this mistake led to an average score 1.89 from a maximum of 4.0 for Problem 4.  Problem 2 was also constructed by following intuitive rules, in particular, “Same A—Same B” [29].

Problem 2

- Angle 2 is greater than angle 1. ✓
- Angle 1 is equal to angle 2.

Problem 3. The rectangle A_1_ is rotated 90° to obtain the rectangle A_2_.

Indicate the correct alternative:

- The area of rectangle A_1_ is different from the area of triangle A_2_.
- The areas of both rectangles are equal. ✓

Problem 4. The rectangle A is wound in the ways indicated in the figures to construct the cylinders B and C.

Indicate the correct alternative:

- The volume of cylinder C is different from the volume of cylinder B.
- The volumes of both cylinders are equal.

Example 3 (mathematical sets and conceptual metaphors). Lakoff and Nunez [72] stated that the concept of mathematical set is internalized using the image-schema CONTAINER [73]. The container is the source domain, whereas the mathematical set is the target domain of a conceptual metaphor. The various properties of the set are conceptualized through the metaphor “sets are containers.” Thus, for example, “an element belongs to the set” is conceptualized as “the element is inside the container,” and “A is a subset of B” is conceptualized as “The container A is inside the container B.” In contrast, Fischbein and Baltsan [74] postulated that the mathematical set is conceptualized as an image-schema COLLECTION. Although Fischbein does not use the term metaphor, using *tacit model* instead, there are no major differences if we assume “sets are collections” as a new metaphor about the mathematical sets. We focus on common mistakes to detect the use of this type of conceptualizations. For this, we build Problem 6 to Problem 10 (see the supplementary material). In Problem 5, for example, the conceptualization of mathematical sets as collections was difficult in practice. It is counterintuitive to think of a collection of elements without any elements. For this reason, we should expect incorrect answers in cases of conceptualizations through collections. The average score on this problem was 0.42 out of a maximum of 4.0.

Problem 5. Given the proposition,

(B.1) $$\begin{array}{c} P=\text{“}with\;the\;solutions\;of\;the\;system\;of\;equations\;\\ 4x+3y=2,\\ 4x+3y=-2, \end{array}$$

it is possible to build a set,” indicate the correct alternative:

- *p* is false.
- *p* is true. ✓

## Supplementary Materials

Supplementary Materials All mathematical problems.
