# Supplementary material for: Mining EEG with SVM for Understanding Cognitive Underpinnings of Math Problem Solving Strategies
Source: Behav Neurol. 2018 Jan 11;2018:4638903. doi: 10.1155/2018/4638903 (PMC5835340; doi:10.1155/2018/4638903)
Supplement: Supplementary Materials — All mathematical problems. [file 4638903.f1.pdf]

Supplementary Materials: All mathematical problems.

Problem 1

Consider a pentagon and a hexagon. All sides of the pentagon are equal, all sides of the hexagon are equal. The sides of the pentagon are equal to the sides of the hexagon. Indicate the correct alternative:

a. Angle 2 is greater than angle 1. ✓

b. Angle 1 is equal to angle 2.

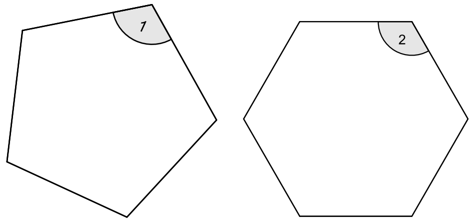

Problem 2

In the figure, the area of the triangle BCE is **S**. The area of the square $ABCD$ is:

a. $2S$.

b. $2\sqrt{3}S$. ✓

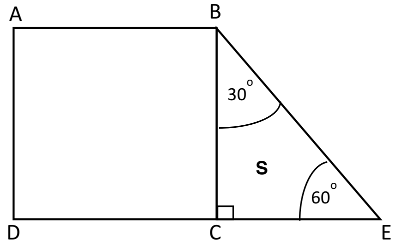

Problem 3

Given the propositions:

$p =$ "With the numbers 2,4,6,8,10,12 it is possible to build a Set".

$q =$ "With the numbers 2,3,7,5,-1,3,6 it is possible to build a Set".

Indicate the correct alternative:

a. p and q are true. ✓

b. Only p is true.

Problem 4

Given the proposition:

$p =$ "With the solutions of the system of equations $\begin{cases} 4x+3y &= 2 \\ 4x+3y &= -2 \end{cases}$ it is possible to build a Set".

Indicate the correct alternative:

a. p is false.

b. p is true. ✓

Problem 5

The equation $x^2 - 5x + 6 = 0$ has as solutions the values 2 and 3. Given the following propositions:

$p =$ "The even solution of the equation forms a Set".

$q =$ "The odd solution does not form a Set".

$r =$ "The solutions of the equation form a Set".

Indicate the correct alternative:

a. Only r is correct.

b. Only q and r are correct.

c. Only p, r are correct. ✓

Problem 6

Indicate the correct alternative:

a. The number of elements (cardinal) contained in the closed interval $[-1, 1]$ of the number line is 3.

b. The number of elements (cardinal) contained in the open interval $(-1, 1)$ of the number line is 1.

c. Both intervals $[-1, 1]$ and $(-1, 1)$ have the same number of elements. ✓

Problem 7

Given the sets A, B, C and the proposition $p =$ "$A \cup C = B \cup C$ implies that $A = B$". Indicate the correct alternative:

a. The proposition p is true for all sets A, B and C.

b. The proposition p is false for any sets A, B and C.

c. The proposition is only true for certain sets. ✓

Problem 8

If x, y are negative numbers, which of the following numbers are also negative?

I. $-(x + y)$.

II. $-2x - 2y$.

III. $-x^2 - y^2$.

a. Only II.

b. Only III. ✓

c. Only I and II.

Problem 9

The rectangle A_1 is rotated 90^0 to obtain the rectangle A_2

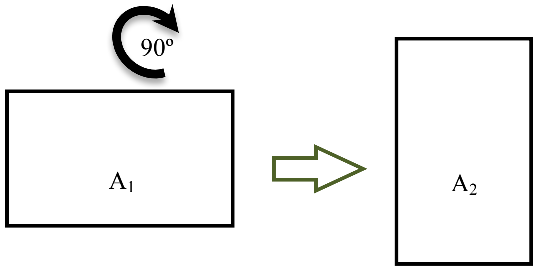

Indicate the correct alternative:

a. The area of rectangle A_1 is different from the area of triangle A_2

b. The areas of both rectangles are equal ✓

Problem 10

The rectangle A is wound in the ways indicated in the figures to construct the cylinders B and C:

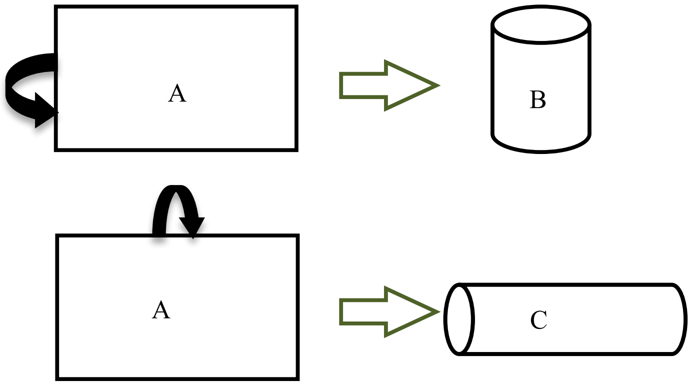

Indicate the correct alternative:

a. The volume of cylinder C is different from the volume of cylinder B ✓

b. The volumes of both cylinders are equal

Problem 11

In which case(s) is the dash line, tangent to the solid line in point P?

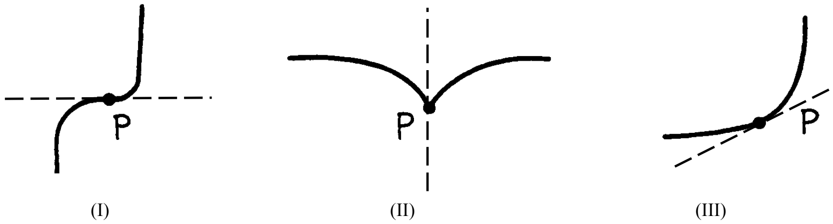

a. I.

b. III.

c. In all cases ✓

Problem 12

If x and y are positive numbers such that $x > y$, it follows correctly that:

I. $\frac{1}{x} > \frac{1}{y}$

II. $-x < -y$

III. $x - y > 0$

a. Only I is correct

b. Only I and II are correct

c. Only II and III are correct ✓

Problem 13

If r, s, t, w are real numbers such that: $r < 0$, $s < 0$, $t < s$, and $w > 0$. Which one(s) of the following statements is(are) true?

I. $w > r$

II. $w \cdot t < r \cdot t$

III. $w \cdot t < r \cdot s$

a. Only I and II are correct

b. Only II and III are correct

c. I, II and III are correct ✓

Problem 14

Let r and s be two real numbers, which one(s) of the following condition(s) is(are) sufficient for $\sqrt{\frac{r}{s}}$ to be also a real number.

I. $r \geq 0$ and $s > 0$

II. $r \leq 0$ and $s < 0$

III. $r > 0$ and $s \neq 0$

a. Only I and II are correct ✓

b. Only II and III are correct

c. I, II and III are correct

Problem 15

The sides of a right-angled triangle are a, b and c. Where c represents the length of the hypotenuse and a and b the lengths of the triangle's other two sides. Assume that the lengths of the sides a and b are variable. In order for the area of this triangle to remain constant, it must be observed that:

a. The triangle's perimeter must be constant.

b. One side must be directly proportional to the other.

c. One side must be inversely proportional to the other.✓

Problem 16

A pencil and a notebook cost 150 pesos together. If the notebook costs 100 pesos more than the pencil, how much does each one cost?

a. Notebook 100 and pencil 50.

b. Notebook 125 and pencil 25.✓

Problem 17

The figure is the graph of the function derivative $y = f'(x)$

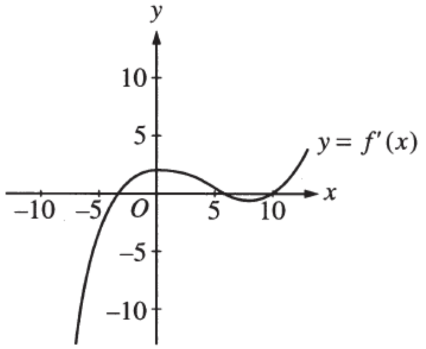

Which of the following graphs could be that of function $y = f(x)$?

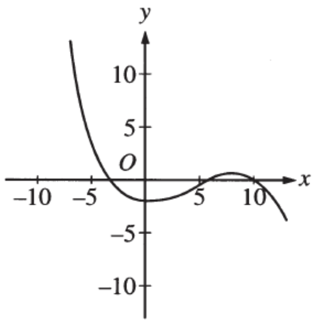

(**A**)

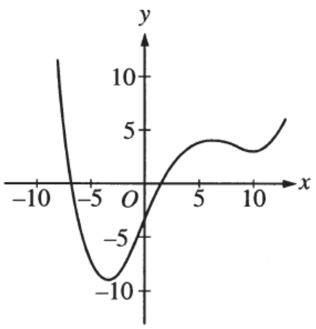

(**B**)

a. (A)

b. (B)✓

Problem 18

Which of the following graphs represents the curve $\{(x,y) = (\sin(t), \cos(t), -\frac{\pi}{2} \leq t \leq 0\}$ in the XY plane?

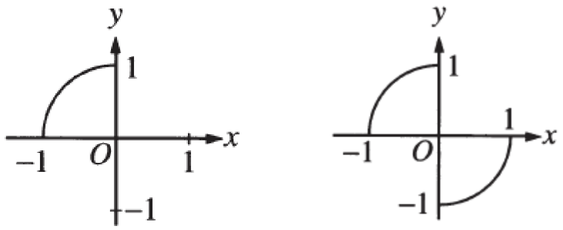

(A) (B)

a. (A)✓

b. (B)

Problem 19

Given the expression:

$$a^b = \sqrt{a\sqrt{a\sqrt{a}}}$$

the value of b is:

a. $\frac{1}{8}$

b. $\frac{3}{8}$

c. $\frac{7}{8}$ ✓

Problem 20

If a is directly proportional to b, it must satisfy

a. $a^2 + b^2$ is proportional to $a^2 - b^2$. ✓

b. $a^2 + b^2$ is inversely proportional to $a^2 - b^2$.
